# Impact of exosomes derived from adipose stem cells on lymphocyte proliferation and phenotype in mouse skin grafts

**DOI:** 10.20517/evcna.2024.52

**Published:** 2025-03-07

**Authors:** Xinqiang Li, Xueteng Wang, Hailun Cai, Ye Wang, Xin Zhou, Bin Wu, Jinzhen Cai, Dahong Teng

**Affiliations:** ^1^Organ Transplantation Center, Affiliated Hospital of Qingdao University, Qingdao 266100, Shandong, China.; ^2^Institute of Organ Donation and Transplantation, Medical College of Qingdao University, Qingdao 266100, Shandong, China.; ^3^Organ Transplant Center, Fujian Medical University Union Hospital, Fuzhou 350000, Fujian, China.; ^4^Pathology Department, the Affiliated Hospital of Qingdao University, Qingdao 266100, Shandong, China.; ^#^Authors contributed equally.

**Keywords:** Adipose-derived stem cells, exosome, mouse skin graft, transplant immunity

## Abstract

**Aim:** Exosomes derived from adipose-derived stem cells (ASCs) in mice have been reported to influence immune regulation. Yet, the potential immunological effects of ASCs-derived exosomes and their interaction with lymphocytes during transplant immunity remain understudied.

**Methods:** ASCs from BALB/c mice, along with their conditioned culture medium, were collected for the extraction, isolation, and comprehensive characterization of exosomes. Splenic cell suspensions were isolated from BALB/c mice and subsequently processed for downstream analyses. Lymphocytes were isolated via gradient centrifugation and stimulated *in vitro* with the purified exosomes to assess their functional responses. Lymphocyte proliferation was quantified using the CCK8 assay, and the relative frequencies of CD4+ T cells, CD8+ T cells, Treg cells, NK (natural killer) cells, macrophages, B cells, dendritic cells (DCs), and Th17 cells were determined through flow cytometric analysis. Before establishing the skin transplantation model, the mice were administered PBS, 0.5 × 10^8^ exosomes, 1 × 10^8^ exosomes, 1.5 × 10^8^ exosomes, or ASCs via intravenous injection through the tail vein. Seven days after transplantation, the spleens, drainage lymph nodes, and blood samples were harvested for lymphocyte isolation and further downstream analyses.

**Results:** Exosomes derived from ASCs significantly increased the CD4+/CD8+ ratio and Treg cell levels, without inducing any notable changes in Th17 cell content or CTLA-4 protein expression in CD4+ T cells. Compared to the PBS-treated group, both ASC and exosome treatment groups demonstrated an enhanced CD4+/CD8+ ratio, increased Treg cell content, and elevated CTLA-4 protein expression in spleen tissue following skin transplantation, while Th17 cell levels remained unaffected. Compared to the ASC treatment group, the exosome group exhibited a higher CD4+/CD8+ ratio and Treg cell levels, alongside a reduced proportion of PD-1+ Treg cells and lower CTLA-4 protein expression in CD3+CD4+ T cells. No significant differences were observed in the proportions of NK cells, macrophages, B cells, and DCs in the spleens across all treatment groups. In peripheral blood, an increased proportion of CD3+ T cells, macrophages, and DCs was detected, accompanied by a reduced proportion of NK cells and B cells. In the draining lymph nodes, no significant changes were observed in the proportions of CD3+ T cells and B cells, while macrophages, NK cells, and DCs showed elevated proportions. In the exosome-treated group, mouse grafts exhibited a disorganized and thinner granular layer, accompanied by focal regions of inflammatory cell infiltration. Both exosome and ASC treatments significantly extended the survival of skin grafts.

**Conclusion:** Exosomes derived from ASCs promote lymphocyte proliferation and modulate their phenotypic profiles in mouse skin graft models, effectively extending graft survival.

## INTRODUCTION

Organ transplantation hinges on striking the right equilibrium between transplant rejection and immune tolerance, both central to transplant immunity^[1]^. Post-transplantation, the interplay between immune and parenchymal cells from both the donor and recipient establishes a unique immune microenvironment^[2]^. Transplant rejection manifests when the recipient’s immune cells target the donor’s parenchymal cells, frequently with an influx of CD8+ T cell infiltration^[3,4]^. This reaction is predominantly regulated through immunosuppressive agents. In contrast, immune tolerance is marked by the expansion of CD4+ Treg cells and can manifest without the aid of immunosuppressants^[5]^. Though numerous strategies exist to mitigate transplant rejection and foster immune tolerance, the pervasive use of immunosuppressants may result in unwanted side effects^[6]^. Hence, the search for novel techniques to regulate transplant immunity harmoniously continues.

Adipose-derived stem cells (ASCs) are increasingly being recognized as potent agents for cell-based therapies(such as reducing inflammation and promoting cartilage tissue repair in osteoarthritis, skin rejuvenation and treatment of autoimmune diseases)^[7,8]^, primarily due to their easy accessibility, impressive proliferative capacity, and minimal immunogenicity^[9,10]^. Beyond the cells themselves, exosomes - represent a subtype of small extracellular vesicles (EVs): the diameter of intraluminal vesicles of endosomes is generally smaller than 200 nm - transfer their contents to recipient cells, acting as messengers^[11]^. ASC-derived exosomes, a significant byproduct from ASCs, play pivotal roles in a myriad of biological activities such as angiogenesis, immune modulation, proliferation, and migration^[12,13]^.

Emerging evidence suggests that exosomes, including those derived from ASCs, act as potent mediators of the immune response by carrying a diverse array of bioactive molecules, such as proteins, lipids, and nucleic acids^[14]^. These molecules can be absorbed by target cells and trigger various intracellular processes, leading to altered cellular behavior. Particularly, ASC-derived exosomes might affect T cell responses, potentially through the modulation of pro-inflammatory cytokine secretion, regulation of antigen-presenting cell functions, or direct interaction with T cell receptors^[15]^. The remarkable ability of exosomes to modulate immune responses at the cellular level underscores their sophisticated mechanism of action, enabling precise fine-tuning of immune reactions rather than merely suppressing or enhancing them. Exosomes, as natural delivery vehicles, can be specifically engineered to elicit immune tolerance in transplantation, demonstrating their potential as versatile and effective therapeutic tools^[16]^. It is this nuanced modulation, which, if harnessed correctly, could provide a strategic advantage over traditional immunosuppressants, minimizing adverse effects while maximizing therapeutic benefit.

Notwithstanding the proven immunomodulatory capabilities of ASC-derived exosomes in skin wounds^[17]^, diabetic nephropathy^[18]^, acute colitis^[19]^, and liver fibrosis^[20]^, their function in the realm of transplant immunity remains relatively untapped. This study delves into the influence of ASC-derived exosomes on lymphocyte proliferation and the phenotypic modulation of mouse skin grafts. Our research could underscore the prospective utility of ASC-derived exosomes as immune modulators, charting new therapeutic pathways to harmonize transplant immune responses.

## METHODS

### Experimental material

#### Experimental animals

BALB/c mice, male, SPF grade, purchased from Beijing Huafukang Biotechnology Co., LTD., License No. SCXK (Beijing) 2019-0008; C57BL/6J mice (extra animals for replacement), male, SPF grade, purchased from Spiff (Beijing) Biotechnology Co., LTD., License No. SCXK (Beijing) 2019-0010; Feeding conditions: temperature 18-26 ℃, humidity: 30%-70%.

#### Experimental reagents

Collagenase I (C8140, Solarbio); Mouse adipose mesenchymal stem cell osteogenic induction solution (PD-025, Prenoxel); Mouse adipogenic mesenchymal stem cells (PD-027, Prenoxel); Mouse adipose mesenchymal stem cell chondrogenic induction solution (PD-026, Prenoxel); CD105 PE (Biolegend 103029); CD34 PE (Biolegend, 128609); CD45 PERCP (Biolegend, 103129); CD90 PE-CY7 (Biolegend, 105307); CD73 FITC (Biolegend, 105307); Saturated Oil Red O staining solution (G1260,Solarbio); Isopropyl alcohol (67-63-0, Tianjin Damao Chemical Reagent Factory); Alizarin red (KGA363-1 keyl); Collagen II (AF0135, Affinity); Antibody Horseradase labeled Goat Anti-Rabbit IgG(H+L) (ZB-2301, Zhongshan Jinqiao); DAB Color development kit (CW0125, CWBIO); Neutral resin (CW0136, CWBIO); Hematoxylin dye solution (AR1180-1, Bosonde); FITC Mouse Anti-Human CD9 (BD, 555371); FITC Mouse Anti-HumanCD63 (BD, 556019); Anti-TSG101 Antibody(ET1701-59,HUABIO); Anti-ALIX Antibody(ET1705-74,HUABIO); Anti-Calnexin Antibody(ET1611-86,HUABIO); Anti-TOMM20 Antibody(ET1609-25,HUABIO); Mouse TGFβELISA kit (MM-0689M2, enzyme-free); Mouse FOXP3 ELISA kit (MM-44741M2, enzyme free); Mouse HGF ELISA kit (MM-0108M2, enzyme free); BCA protein quantitative kit (NCI3225CH, Pierce); PerCP/Cy5.5/Cyanine5.5 anti-mouse CD3 Antibody (Biolegend, 100218); APC anti-mouse CD4 Antibody (Biolegend, 116014); APC/Cyanine7 anti-mouse CD8a Antibody (Biolegend, 100714); RBC Lysis Buffer (10X) (Biolegend, 420301); PE/Cyanine7 anti-mouse IL-17A Antibody (Biolegend, 506922); PE anti-mouse CD4 Antibody (Biolegend, 116006); APC anti-mouse CD152 Antibody (Biolegend, 106310); FITC anti-mouse CD25 Antibody (Biolegend, 101907); FITC anti-mouse CD3 Antibody (Biolegend, 100204); APC anti-mouse CD11c Antibody (Biolegend, 117309); PE anti-mouse NK1.1 Antibody (Biolegend, 108707); Brilliant Violet 421™ anti-mouse CD11b (Biolegend, 101251); Brilliant Violet 605™ anti-mouse F4/80 (Biolegend, 123133); PerCP/Cyanine5.5 anti-mouse CD45 (Biolegend, 103131); PE/Cyanine7 anti-mouse CD19 (Biolegend, 115519); APC-A750 Zombie NIR™ Fixable Viability Kit (Biolegend, 423105); FOXP3 Monoclonal Antibody (3G3), PE (Invitrogen, MA1-10332); Cell Activation Cocktail (with Brefeldin A) (Biolegend, 423303); True-Nuclear™ Transcription Factor Buffer Set (Biolegend, 424401).

#### Experimental Instruments

CO2 incubator (BPN-80CW, Shanghai Yiheng Scientific Instrument Co., LTD.); Inverted fluorescence microscope (MF53, Guangzhou Mingmei Optoelectronics Co., LTD.); NovoCyte™ Flow cytometry (NovoCyte 2060R, Aisen Biology (Hangzhou) Co., LTD.); Microscope (CX41, OLYMPUS); Electric blast drying box (DHG-9070A, HengScientific Instrument Co., LTD.); Thermostatic incubator (DHP-9054, Shandong Boke Biology); ZetaVIEW, PARTICLE METRIX; Particle size analyzer (N30E, NanoFCM); Multifunctional enzyme label instrument (Varioskan LUX, Thermo); Chemiluminescence Gel Imaging System (ChemiScope 3000mini, CLINX).

### Experimental method

#### Isolation and culture of mouse ASCs

Six-week-old mice were first disinfected by soaking in 75% alcohol for 3 min. They were then positioned on an ice plate within a sterilized environment. From the subcutaneous region of the mice’s groin, white adipocytes were rapidly extracted, ensuring the thorough removal of any visible blood vessels and fibrous tissues. These adipocytes were then subjected to enzymatic digestion at 37 °C for 20 min, undergoing intermittent agitation at 5-min intervals. Upon completion of digestion, the enzymatic reaction was halted by adding a complete medium (containing 20% FBS and 1% Penicillin Streptomycin) supplemented with 10% FBS (2036224C, Gibco). The digested tissue was subsequently passed through a 70 µm cell strainer and the resultant filtrate was centrifuged at 1,000 r/min for 5 min. The supernatant was discarded, and the cell pellet was resuspended in a fresh culture medium. Upon reaching 80%-90% confluence, cells were twice washed with 1 × PBS (KGB5001, Jiangsu KeyGEN BioTECH Corp., Ltd), detached from the culture flask using a 0.25% pancreatin solution enriched with 0.02% EDTA, and centrifuged at 1,000 r/min for 3 min. The cells were then resuspended in medium, an equal amount of medium was added, and the cells were dispensed into new Petri dishes at a dilution ratio of 1:2 and incubated in an incubator.

#### Flow cytometry analysis of mouse ASCs

For the flow cytometry analysis, third-generation ASCs were washed with 1 × PBS and detached using a 0.25% pancreatin solution containing 0.02% EDTA. This was followed by a 3-min centrifugation at 1,000 r/min. The collected cells were resuspended in PBS and distributed into six tubes. One tube was designated as the control, while the others were individually labeled with specific antibodies: CD105 PE, CD34 PE, CD45 PERCP, CD90 PE-Cy7, and CD73FITC, each tube receiving 5 µL of its respective antibody. Tubes were mixed gently and incubated in a darkened environment at room temperature for 20 min. After incubation, cells were washed twice with PBS, with each wash followed by a 5-min centrifugation at 1,500 r/min. Lastly, cells were suspended in 500 µL PBS and analyzed using flow cytometry.

#### Lipogenesis induction in mouse ASCs

Third-generation ASCs were twice washed with 1 × PBS and treated with 0.25% pancreatin supplemented with 0.02% EDTA. Once cells rounded, digestion was halted with culture medium. Cells were then transferred to a 10 mL centrifuge tube and spun at 1,000 rpm for 3 min. After discarding the supernatant, cells were resuspended in culture medium to achieve a concentration of 1 × 10^5^ cells/mL. Subsequently, cells were seeded onto 12-well plates. Upon achieving 70%-80% confluence, cells underwent lipogenic induction (Mouse Adipose Mesenchymal Stem Cell Lipogenic Induced Differentiation Medium, Procell Life Science&Technology Co., Ltd, PD-027): adipogenic differentiation medium A was induced for 3 days followed by adipogenic differentiation medium B was induced for 1 day. On day 14 post-induction, oil red staining was employed for verification.

#### Osteogenic induction in mouse ASCs

Third-generation ASCs were processed as described above, reaching a concentration of 1 × 10^5^ cells/mL. Cells were then seeded onto 12-well plates. Once they reached a confluence of 70%-80%, an osteogenic induction medium (Mouse Adipose Mesenchymal Stem Cells Osteogenic Induced Differentiation Medium, Procell Life Science&Technology Co., Ltd, PD-025) was added. This medium was replenished every 3 days for a duration of 4 weeks. Post-induction, alizarin red staining was utilized for verification.

#### Chondrogenic induction in mouse ASCs

Using the same initial processing steps, third-generation ASCs were adjusted to a concentration of 1 × 10^5^ cells/mL and seeded onto 12-well plates. At 70%-80% confluence, a chondrogenic induction medium (Mouse Adipose Mesenchymal Stem Cells Chondrogenic Induced Differentiation Medium, Procell Life Science&Technology Co., Ltd, PD-026) was introduced. This medium was renewed every 3 days over a 4-week period. Following the induction period, immunohistochemical staining was employed for verification.

#### Oil red O staining procedure

Cells were initially deprived of their culture medium and subsequently fixed using a 4% tissue cell fixative for 30 min. Following fixation, they were thoroughly rinsed with distilled water and infiltrated with 60% isopropyl alcohol. The oil red solution was applied to stain the cells for a span of 10 min, after which it was removed, and differentiation was undertaken using 60% isopropyl alcohol. A final rinse with distilled water came before staining the nuclei with hematoxylin for 1 min. Microscopic examination revealed lipid droplets in cells to exhibit an orange-red color, while the nuclei were blue.

#### Alizarin red staining procedure

The procedure started with the removal of the culture medium from the cells. Cells were then fixed in a 4% tissue fixative solution for 30 min. Post fixation, cells were rinsed with PBS and allowed to air dry to remove excess water. An application of alizarin red dye was then performed for each well, staining for about 40 min. A swift rinse with distilled water followed, post which the stained cells were documented using photography.

#### Immunohistochemical staining procedure

Commencing the procedure, cells underwent three successive PBS rinses, each lasting 3 min. A fixation step using 4% paraformaldehyde was then carried out for 15 min. After three more PBS rinses, the cells were permeabilized using a 0.5% Triton X-100 solution in PBS at room temperature for 20 min. A 3% hydrogen peroxide solution was then introduced for 10 min to block any endogenous peroxidase activity. Another series of PBS rinses followed before the addition of 5% BSA to block non-specific binding, incubating at 37 °C for 30 min. The primary antibody targeting Type II collagen (at a 1:200 dilution) was introduced and incubated overnight at 4 °C. After primary antibody incubation, plates were warmed to room temperature for 45 min and subsequently rinsed with PBS. A secondary antibody application, specifically the HRP-labeled anti-rabbit IgG, followed with an incubation at 37 °C for 30 min. DAB substrate was applied for 5-10 min, with staining intensity monitored under a microscope. The final steps included a rinse, counterstaining with hematoxylin for 3 min, differentiation with hydrochloric acid alcohol, bluing, and a last rinse with tap water. The dehydration, clearing, and mounting processes followed, making the cells ready for microscopic examination.

#### Extraction of exosomes by ultracentrifugation from mouse adipose tissue stem cells

The process begins with collecting supernatants and centrifuging at different speeds to progressively purify the exosomes. Starting at 2,000 × *g* to settle larger cells and debris, the process advances to 10,000 × *g* to remove large vesicles. The supernatant is then filtered using a 0.45 μm membrane. Subsequent ultracentrifugation steps at 100,000 × *g* are employed to pellet the exosomes. Finally, the pellet is resuspended in a small volume of 1 × PBS and stored at -80 °C.

#### Identification of exosomes from mouse adipose tissue stem cells

Transmission electron microscopy (TEM): A drop of exosome suspension is placed on a copper grid and allowed to settle. After the excess liquid is removed, the sample is negatively stained with uranium dioxyacetate and examined under the electron microscope at 100 kv.

Particle Size Analysis: Exosome samples are thawed, diluted, and subjected to Nanoparticle Tracking Analysis (NTA) to ascertain particle size distribution.

Fluorescence Labeling and Nanoflow Detection: Exosome samples are first labeled with fluorescent antibodies specific to exosomal markers (CD9 and CD63). Following labeling, the samples undergo ultracentrifugation to wash away unbound antibodies, and the pellet is resuspended in 1 × PBS.

Western Blot Analysis: Using a 12% SDS-PAGE gel, protein samples from adipose tissue-derived stem cells and exosomes from mouse adipose tissue-derived stem cells are electrophoresed and transferred onto a PVDF membrane. After blocking with 5% skim milk TBST, the membrane is incubated with a primary antibody overnight, followed by a secondary antibody. Post washing, TSG101, Alix, Calnexin, and TOM20 proteins are detected using ECL reagents and imaged for analysis.

#### Extraction and determination of protein concentration from exosomes of mouse adipose tissue stem cells

The exosomal proteins are extracted using RIPA lysate and lysed on ice. The protein concentration is subsequently determined using the BCA method, wherein the absorbance of the samples is measured at OD562 nm, and the concentration deduced from a standard curve.

#### Western blot of exosome protein from mouse adipose tissue-derived stem cells

Using a 12% SDS-PAGE gel, protein samples from adipose tissue-derived stem cells are electrophoresed and transferred onto a PVDF membrane. After blocking with 5% skim milk TBST, the membrane is incubated with a primary antibody overnight, followed by a secondary antibody. Post washing, PEG-2 proteins are detected using ECL reagents and imaged for analysis. A total of 293 cells were used as a control.

#### Exosome ELISA detection of mouse adipose tissue-derived stem cells

ELISA strips are prepared, and both standard and sample solutions are loaded. After the addition of an HRP-labeled detection antibody and incubation, the plate is washed and substrates are added. Following another incubation and the addition of termination solution, absorbance is read at 450 nm to quantify TGF-β, FOXP3 and HGF protein concentrations.

#### Preparation of C57BL/6J mouse spleen single cell suspension

After extracting the spleen from a 6-week-old male C57BL/6J mouse, it is finely cut and ground on a cell filter screen using a syringe piston in the presence of PBS. The resulting cell suspension is filtered to ensure single-cell consistency. Following centrifugation at 250 g for 10 min, cells are resuspended in PBS or a cell cleaning solution, centrifuged again, and then finally suspended at a density of approximately 1-10 × 10^6^ cells/mL.

#### Flow cytometry

Cell suspension (100 uL) is stained with CD45, CD11c, CD3, CD11b, F4/80, NK1.1, CD19, CD4, CD8, CD25, CD152, PD-1 and Fixable Viability Kit. After antibody binding, cells are fixed and permeabilized, then stained with FOXP3 and IL-17A. Cells are washed and resuspended in PBS for final analysis in a flow cytometer.

#### Animal treatment and skin graft model construction

Experimental mice were divided into five groups: PBS control (*N* = 6); 0.1 mL of exosomes with 0.5 × 10^8^ particles Exosome injection (*N* = 8); 0.1 mL of exosomes with 10^8^ particles Exosome injection (*N* = 8); 0.1 mL of exosomes with 1.5 × 10^8^ particles Exosome injection (*N* = 8) and ASCs injection (*N* = 8). There were three observation groups, PBS control (*N* = 6), 0.1 mL of exosomes with 10^8^ particles Exosome injection (*N* = 8) and ASCs injection (*N* = 8). C57BL/6J mice received tail vein injections of 0.1 mL PBS, or 0.1 mL ASCs at a concentration of 5 × 10^6^/mL. After 24 h, donor BALB/c mice and recipient C57BL/6J mice were anesthetized. Skin areas of 1.5 cm × 1.5 cm were marked near the spine’s head region for both donor and recipient. The donor’s skin was cut to the superficial fascia level and transplanted onto the recipient after making a similar incision. The graft was sutured in place with 5-0 silk thread. Graft conditions, such as color, texture, shedding, and hair growth, were monitored daily. A graft was deemed rejected if it hardened or crusted, whereas it was considered successful if it was soft or showed hair growth. From the day of transplantation, a daily intraperitoneal injection of cephalosporin (1 mg/kg) was administered.

#### Analysis of immune tolerance status of recipients

On the seventh day post-transplantation, C57BL/6J mice from all groups were euthanized. Spleen tissue was harvested and processed to form a single-cell suspension suitable for lymphocyte isolation. Using the CCK8 assay, lymphocyte proliferation rates were determined. Flow cytometry was employed to ascertain the percentages of various T cell populations, specifically CD3+CD4+T cells, CD3+CD8+T cells, Treg cells (characterized by CD3+CD4+CD25+FOXP3+ markers), PD-1+Treg cells (characterized by CD3+CD4+CD25+FOXP3+PD-1+ markers), and Th17 cells (identified by CD3+CD4+IL-17A+ markers). Additionally, the expression levels of the CTLA-4 protein within CD3+CD4+T cells were examined. The grafted tissues were paraffin-embedded, sectioned, and subsequently stained with hematoxylin and eosin (HE). Using the Banff scoring system, these stained sections were then assessed to determine the presence and severity of graft rejection.

#### Donor-specific detection of lymphocyte immune regulation in transplant recipients post-operation

Seven days post-transplantation, C57BL/6J mice from each experimental group were euthanized. Spleen tissue was extracted and processed to produce a single splenic cell suspension. To stimulate the cells, before establishing the skin transplantation model, the mice were administered PBS, 1 × 10^8^ exosomes and ASCs via intravenous injection through the tail vein. Following this, lymphocyte proliferation was gauged using the CCK8 assay.

### Statistical analysis

The data obtained were subjected to statistical analysis using the SPSS19.0 software. All experimental procedures were performed in triplicate, with the quantitative outcomes represented as the mean ± standard deviation (X ± S). A one-way analysis of variance was employed to draw quantitative comparisons among the various groups. When engaging in pairwise comparisons, the LSD (Least Significant Difference) method was utilized. A significance level was set at *P* = 0.05. For graphical representation of the data, Graphpad 8.0 software was employed. Single-cell analysis was performed with R software (version 4.2.3).

## RESULTS

### Characterization of mouse ASCs in single-cell RNA-seq

**Figure 1:**
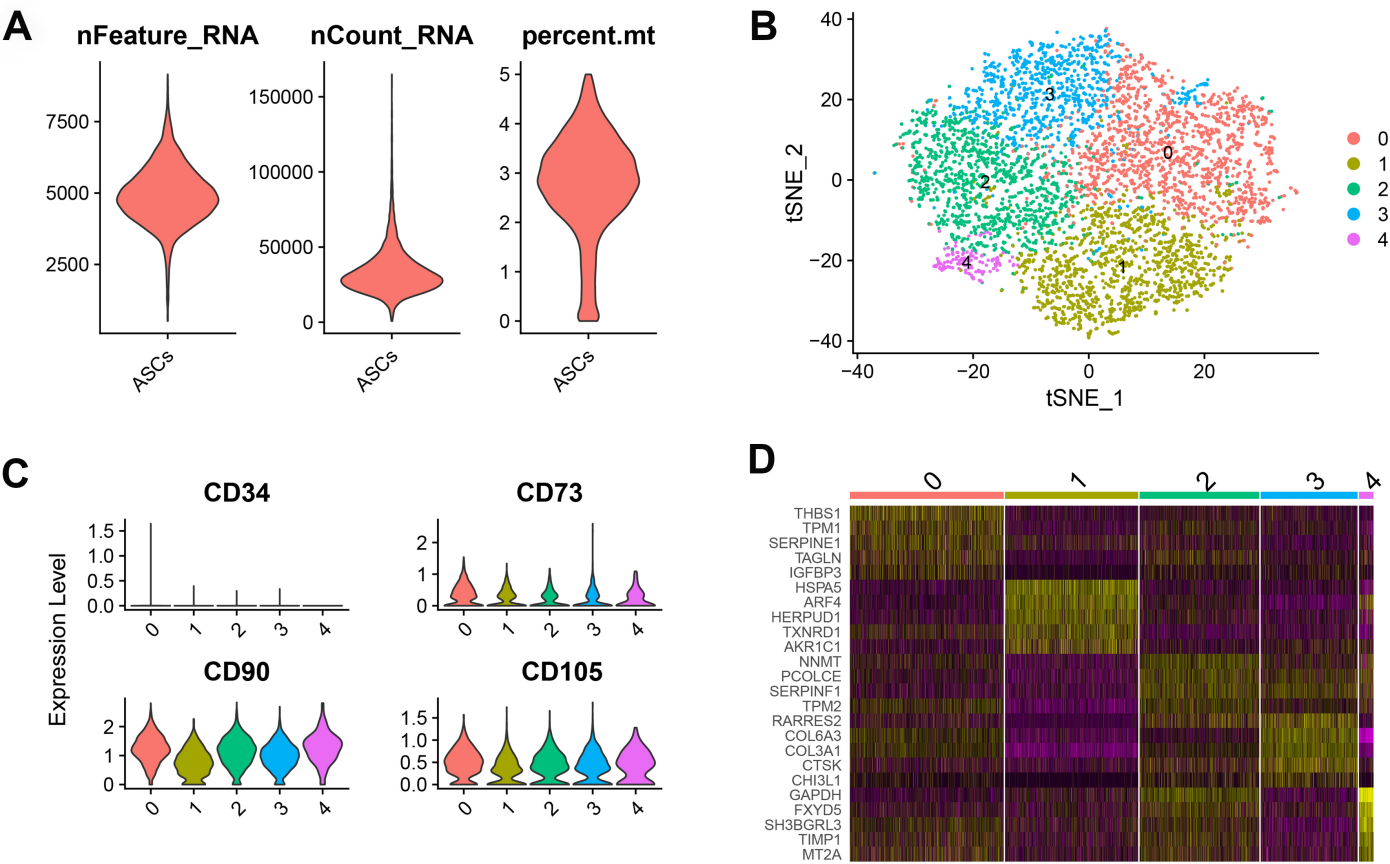
Single-cell RNA-seq of mouse ASCs. (A) Violin plots showed the quality control using nFeature_RNA, nCount_RNA, and percent.mt (refers to the number of genes, UMIs, and the percentage of mitochondrial genes, respectively); (B) TSNE plot showed the distribution of 5 clusters for all the mouse ASCs; (C) Violin plots showed the expression of CD34, CD73, CD90 and CD105 among 5 clusters; (D) Heatmap showed the top 5 markers (differentially expressed genes) of each cluster. ASCs: Adipose-derived stem cells.

Since single-cell analysis showed more accurate and complex data for biology, to further characterize the components of mouse ASCs with distinct features, we performed single-cell RNA analysis with single-cell data from GEO datasets^[21]^ using our previous methods^[22]^. After data filtering, we acquired 5,246 high-quality mouse ASCs [Figure 1A]. Clustering analysis showed 5 distinct clusters from mouse ASCs [Figure 1B]. Gene expression analysis indicated all the clusters exhibited a high expression of CD73, CD90, and CD105 and a low expression of CD34 [Figure 1C]. We further defined these 5 clusters [Figure 1D and Supplementary Table 1], including THBS1+ mouse ASCs (THBS1, TPM1, SERPINE1, TAGLN, and IGFBP3), HSPA5+ mouse ASCs (HSPA5, ARF4, HERPUD1, TXNRD1, and AKR1C1), NNMT+ mouse ASCs (NNMT, PCOLCE, SERPINF1, TPM2, and RARRES2), COL6A3+ mouse ASCs (COL6A3, COL3A1, RARRES2, CTSK, and CHI3L1), and GAPDH+ mouse ASCs (GAPDH, FXYD5, SH3BGRL3, TIMP1, and MT2A).

### Isolation, culture and identification of mouse ASCs

**Figure 2:**
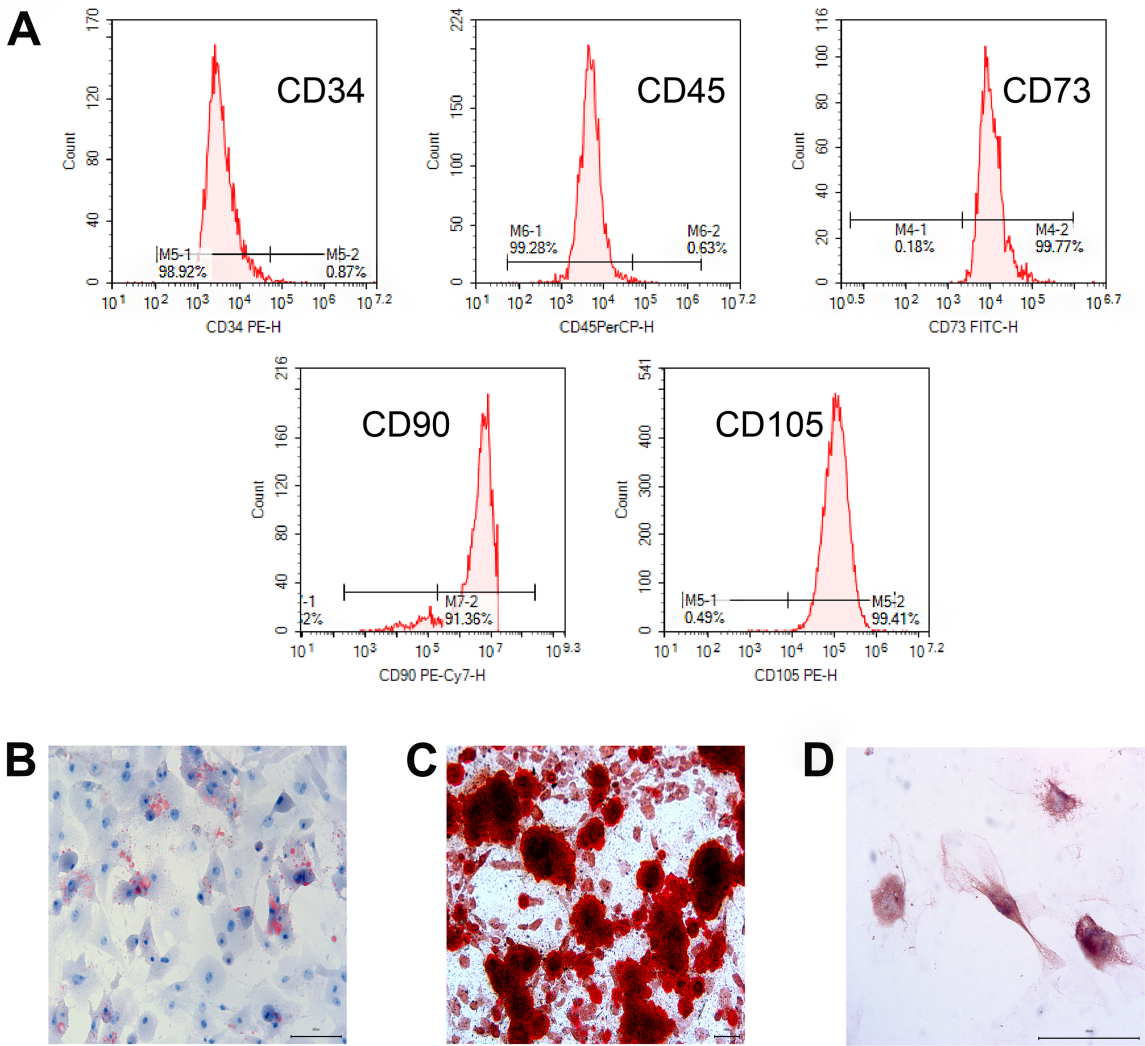
Identification of mouse ASC cells. (A) The expressions of CD34, CD45, CD73, CD90, and CD105 in the isolated mouse ASCs were detected by flow cytometry; (B-D) Induction and identification of adipogenesis, osteogenesis, and chondrogenesis of mouse ASC cells. (B) Oil red O staining (200×); (C) Alizarin red staining (100×); (D) Immunohistochemistry (400×). ASC: Adipose-derived stem cell.

ASCs were isolated from mouse adipose tissue through repeated adherence, and third-generation ASCs were selected due to their robust growth. Flow cytometry revealed positive expressions of CD73, CD90, and CD105 at 99.77%, 91.36%, and 99.41%, respectively, whereas CD34 and CD45 expressions were below 1%, confirming successful mouse ASC isolation [Figure 2A].

We assessed mouse ASCs’ multiline differentiation capability using different induction solutions. Lipogenesis was confirmed using oil red O staining [Figure 2B], osteogenesis by alizarin red staining indicating calcium salt deposition [Figure 2C], and chondrogenesis via significant type II collagen expression as observed through immunohistochemical staining [Figure 2D].

### Extraction and identification of mouse ASC exosomes

**Figure 3:**
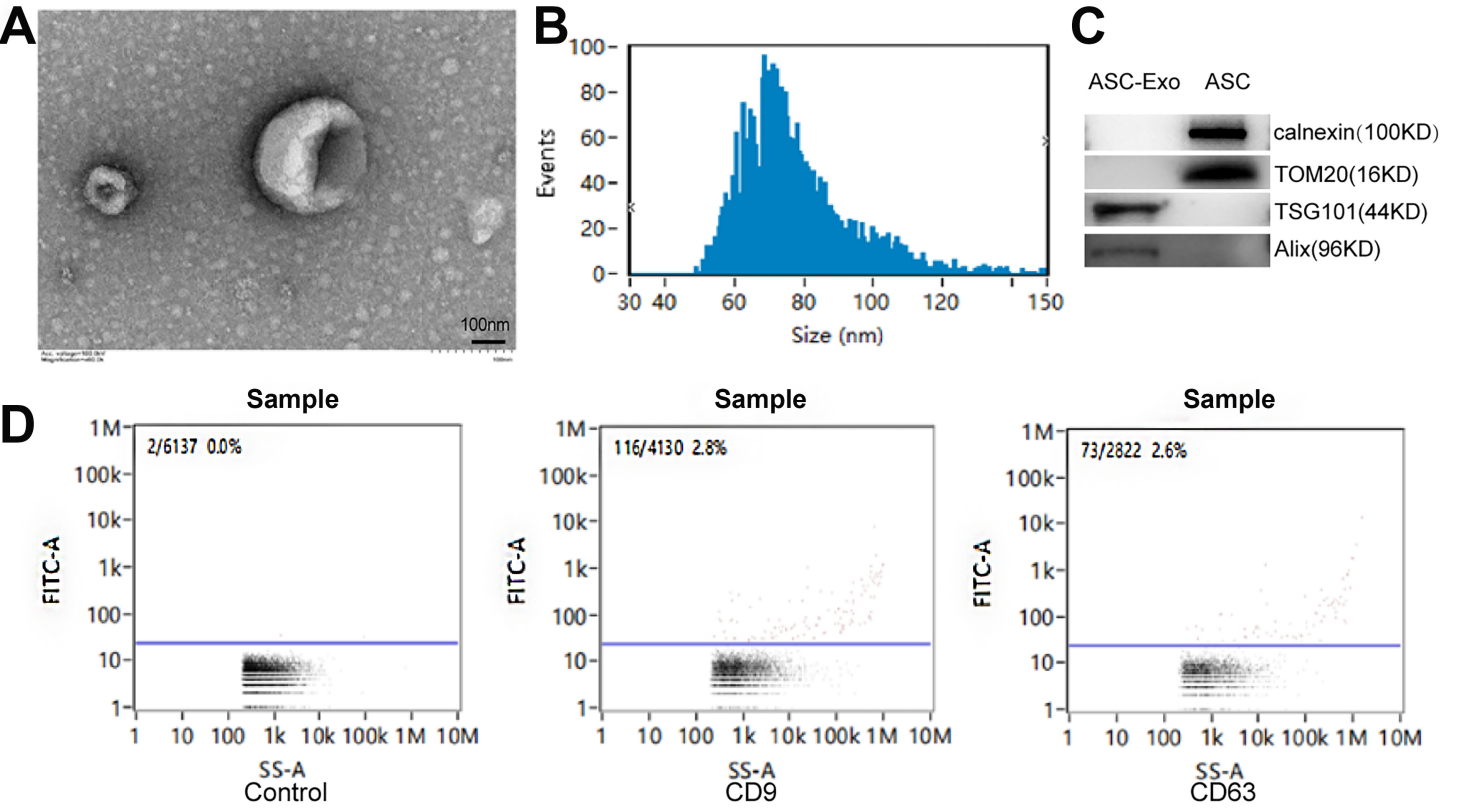
Identification of exosomes by mouse ASCs. (A) Transmission electron microscopy; (B) NTA particle size identification; (C) Western blot analyses of exosome markers such as TSG101 and Alix, and mouse ASCs-related markers calnexin and TOM20; (D) Stream identification. ASCs: Adipose-derived stem cells; NTA: Nanoparticle Tracking Analysis.

Exosomes were harvested from the ASC culture medium via overspeed centrifugation. TEM illustrated their morphology [Figure 3A], while NTA delineated their size distribution [Figure 3B]. Western blot results showed that the exosomal surface marker proteins TSG101 and Alix were all expressed but negative for the mouse ASCs marker proteins calnexin and TOM20 [Figure 3C]. Exosomes exhibited an average size of 78.35 nm, and CD9 and CD63 were expressed positively, confirming successful extraction [Figure 3D].

**Figure 4:**
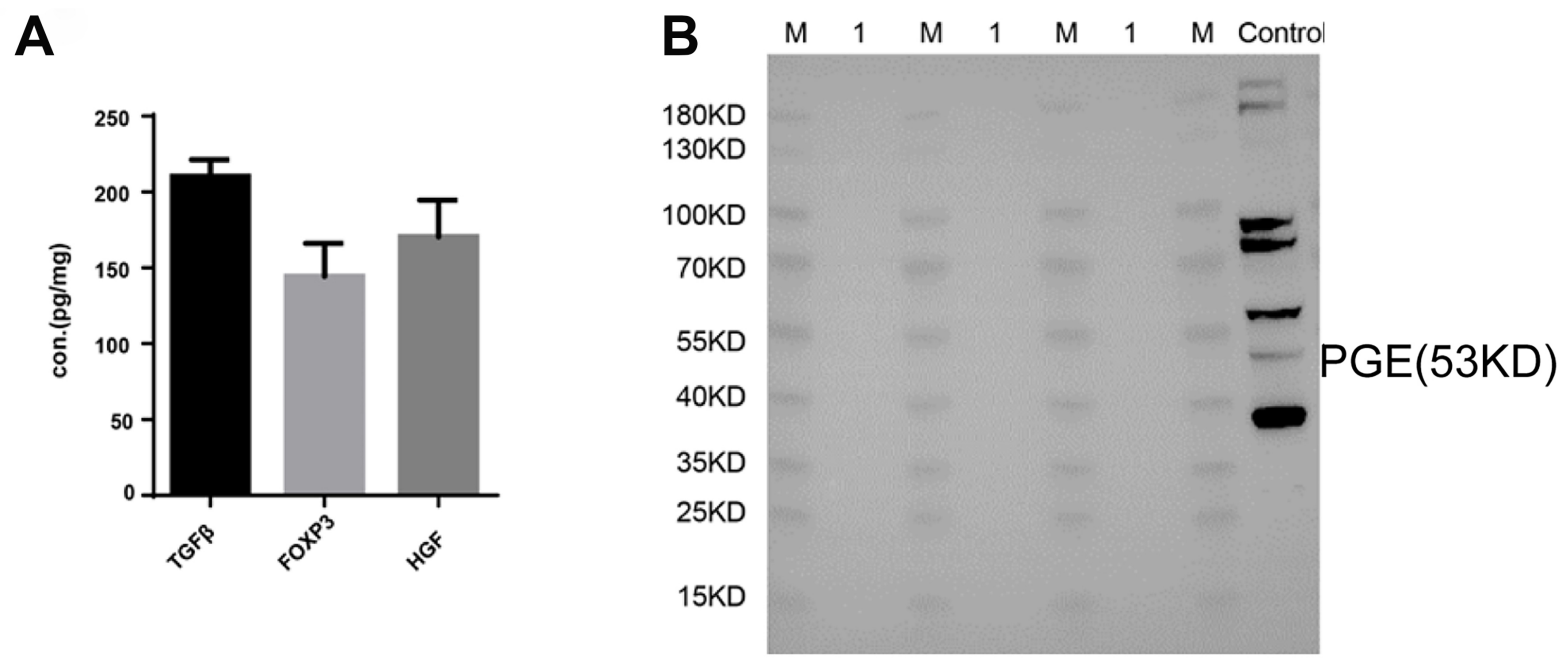
Detection of regulatory cytokines in ASCs exosomes. (A) ELISA to detect TGF-beta, FoxP-3, and HGF in ASC exosomes; (B) The expression of PGE-2 in exosomes of ASCs was detected by Western blot. note: 10 μg/lane, M: Marker; 1: murine adipose-derived mesenchymal stem cell exosomes; Control: 293 cells. ASCs: Adipose-derived stem cells.

ELISA results demonstrated FOXP-3, TGF-β, and HGF expressions in ASCs exosomes with concentrations around 150 pg/mg [Figure 4A]. Western blotting, however, did not detect PGE-2 expression in the exosomes [Figure 4B].

### Mouse ASC exosomes prolong skin graft survival.

**Figure 5:**
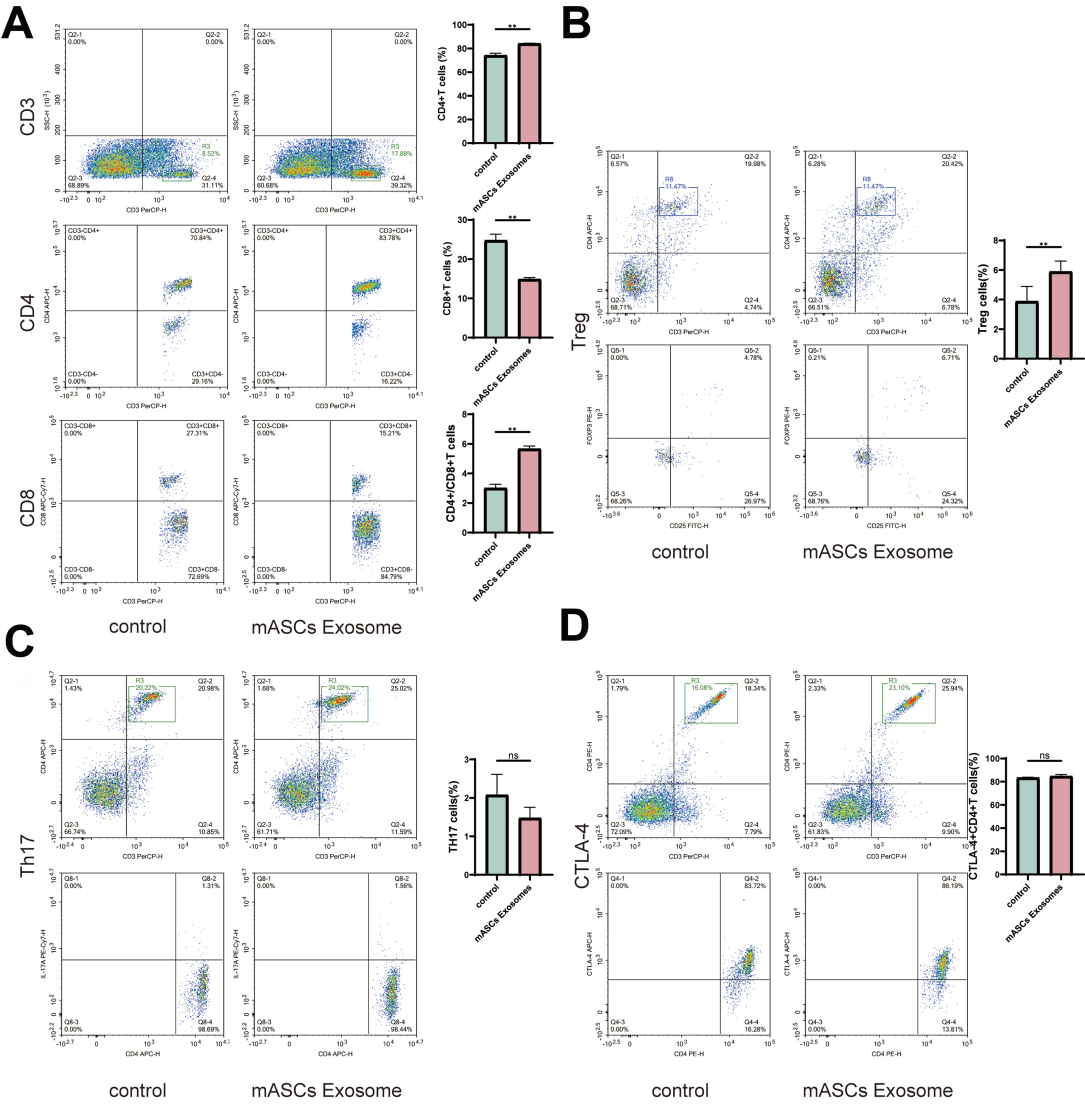
Flow cytometry of CD3+CD4+T cells, CD3+CD8+T cells, Treg cells, Th17 cells, and CD4+CTLA-4+T cells showed different expression for mouse ASC Exosome. (A) Changes in the proportion of CD3+CD4 T cells, CD3+CD8+ T cells, and CD4/CD8 ratio in the control and mASCs Exosome-treated groups; (B) Changes in the proportion of Treg cells in control and mASCs exosome-treated groups; (C) Changes in the proportion of Th17 cells in control and mASCs exosome-treated groups; (D) Changes in the proportion of CD4+CTLA-4+ T cells in control and mASCs exosome-treated groups. ASCs: Adipose-derived stem cells; mASCs: mouse adipose-derived stem cells. ^*^P < 0.05; ^**^P < 0.01

Flow cytometry revealed that, compared to untreated lymphocytes, mouse ASC exosomes elevated the CD4+/CD8+ ratio and Treg cell content significantly [Figure 5A and B]. Th17 cell content remained unchanged, and CTLA-4 protein expression in CD3+CD4+T cells showed no significant alteration [Figure 5C and D].

**Figure 6:**
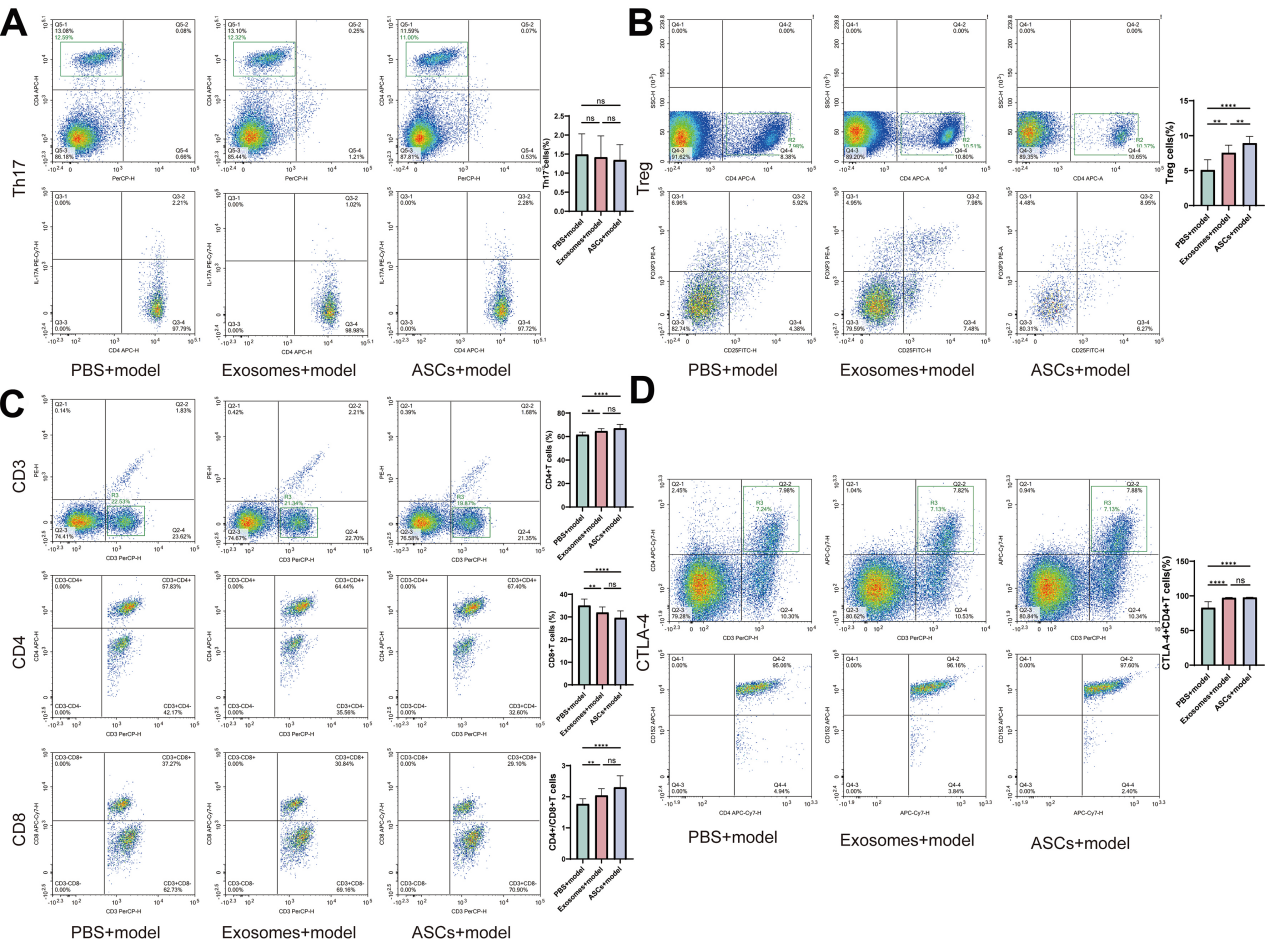
Flow cytometry of CD3+CD4+T cells, CD3+CD8+T cells, Treg cells, Th17 cells, and CD3+CD4+T cells showed different expression of CTLA-4 protein. (A) Changes in the proportion of Th17 cells in the model treated by PBS, Exosomes, and ASCs; (B) Changes in the proportion of Treg cells in the model treated by PBS, Exosomes, and ASCs; (C) Changes in the proportion of CD3+CD4T cells, CD3+CD8+T cells, and CD4/CD8 ratio in the model treated by PBS, Exosomes, and ASCs; (D) Changes in the proportion of CD4+CTLA-4+T cells in the model treated by PBS, Exosomes, and ASCs. ASCs: Adipose-derived stem cells. ^*^P < 0.05; ^**^P < 0.01; ^***^P < 0.001; ^****^P < 0.0001

In skin-grafted mice, flow cytometry analysis indicated that both mouse ASCs and exosomes, when compared to the PBS control, increased the CD4+/CD8+ T cell ratio, Treg cell content, and CTLA-4 protein expression in CD3+CD4+ T cells. There were no significant changes in Th17 cell content. Notably, exosomes further amplified these effects compared to mouse ASC treatment alone [Figure 6A-D].

**Figure 7:**
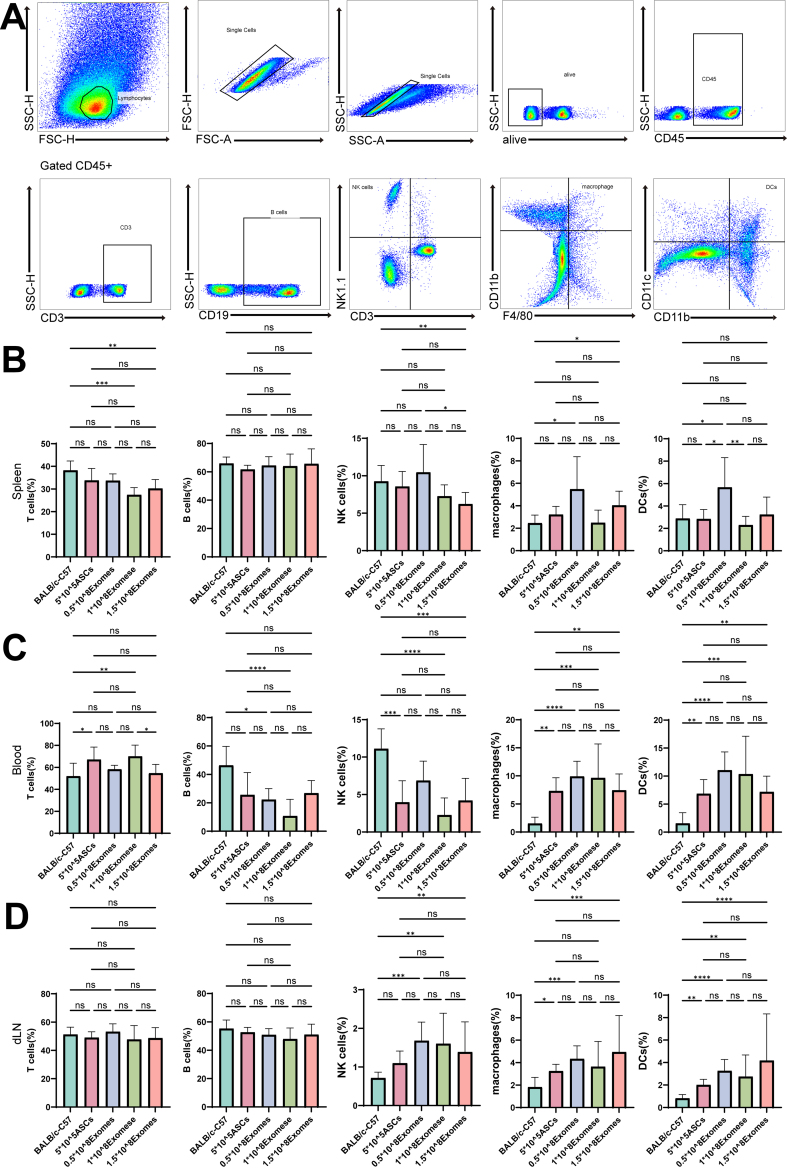
Flow cytometry of CD3+T cells, B cells, NK cells, DC cells, and macrophages. (A) Flow cytometry gating strategy for cutaneous lymphoid subsets; (B) Changes in the proportions of T cells, B cells, NK cells, DC cells, and macrophages in the spleen in models treated with PBS, 0.5 × 10^8^ Exosomes, 1 × 10^8^ Exosomes, 1.5 × 10^8^ Exosomes, and ASCs; (C) Changes in the proportions of T cells, B cells, NK cells, DC cells, and macrophages in the blood in models treated with PBS, 0.5 × 10^8^ Exosomes, 1 × 10^8^ Exosomes, 1.5 × 10^8^ Exosomes, and ASCs; (D) Changes in the proportions of T cells, B cells, NK cells, DC cells, and macrophages in the dLN in models treated with PBS, 0.5 × 10^8^ Exosomes, 1 × 10^8^ Exosomes, 1.5 × 10^8^ Exosomes, and ASCs. DCs: Dendritic cells; NK cells: natural killer cell; ASCs: adipose-derived stem cells. ^*^P < 0.05; ^**^P < 0.01; ^***^P < 0.001; ^****^P < 0.0001.

Similarly, no significant differences were observed in the proportions of other immune cells, including macrophages, dendritic cells (DCs), natural killer (NK) cells, and B cells, within the spleen across the control, ASC, and exosome treatment groups. In peripheral blood, the proportion of T cells increased, while the proportions of B cells, NK cells, DCs, and macrophages were notably reduced. In the draining lymph nodes, no significant changes were observed in the proportions of T cells and B cells; however, NK cells, macrophages, and DCs exhibited increased proportions. [Figure 7A-D].

**Figure 8:**
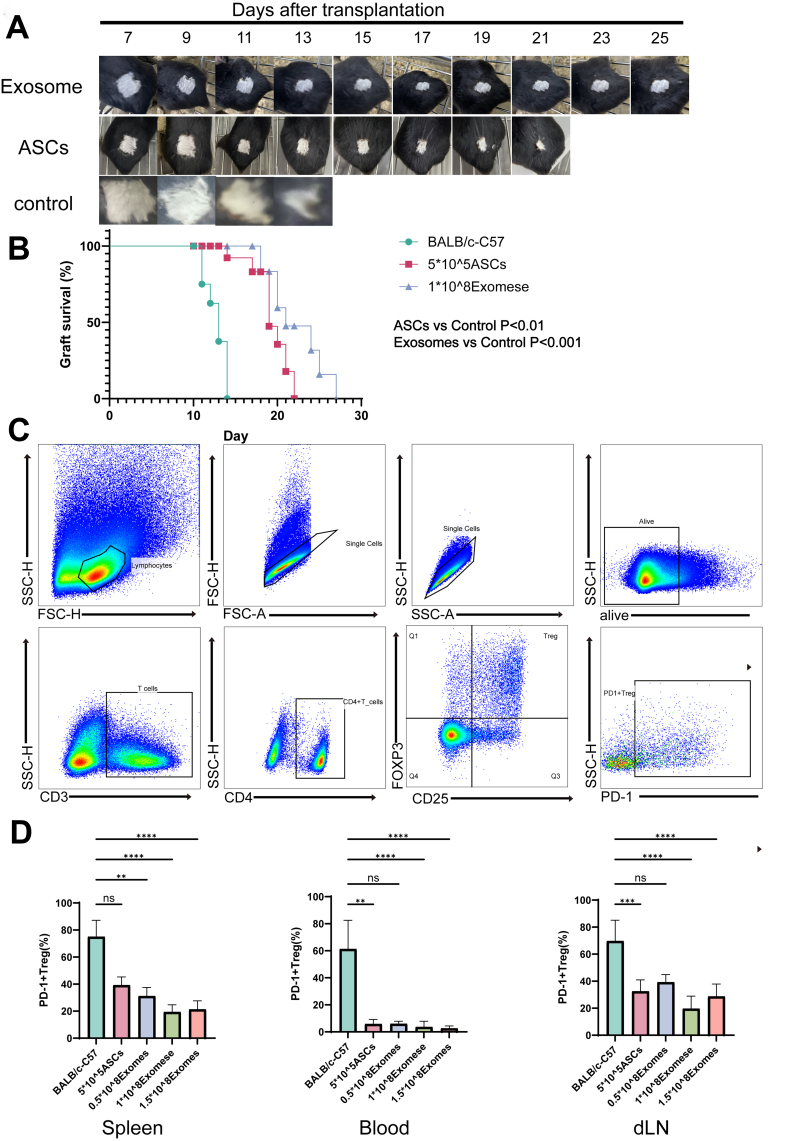
Exosomes and ASC therapy prolong the survival of skin grafts. (A) Skin allograft appearance of Exosomes + model, ASCs + model, and control mice from day 7 to 25 post-transplantation; (B) The graft survival rate in Exosomes + model, ASCs + model, and control mice within 25 days post-transplantation (*n* = 8); (C) Flow cytometry gating strategy for PD-1+Treg subsets; (D) Changes in the proportions of PD-1+Tregs in the spleen in models treated with PBS, 0.5 × 10^8^ Exosomes, 1 × 10^8^ Exosomes, 1.5 × 10^8^ Exosomes, and ASCs. ASCs: Adipose-derived stem cells. ^*^P < 0.05; ^**^P < 0.01; ^***^P < 0.001; ^****^P < 0.0001.

Compared to the control group, PD-1 expression on Tregs was significantly reduced in the spleen following ASC and exosome treatment. Similar reductions in PD-1 expression were also observed in peripheral blood and draining lymph nodes. As key immunomodulatory cells, Tregs exhibited reduced depletion and an increased proportion following ASC and exosome treatment, which collectively contributed to prolonged skin graft survival. [Figure 8A-D].

### Histopathological changes in skin graft model mice

**Figure 9:**
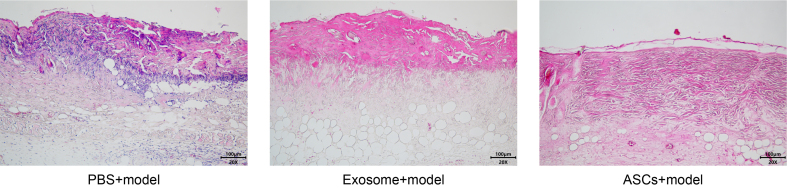
Skin pathology of mice in skin graft model detected by HE staining (×200). ASCs: Adipose-derived stem cells; HE: hematoxylin and eosin.

Histological examination of mouse grafts in the PBS control showed prominent inflammatory cell infiltration and graft necrosis. The exosome group exhibited a disrupted, thinner granular layer with localized inflammatory cell infiltration. Conversely, the ASCs group displayed pronounced stratum corneum thickening with hyperkeratosis, stratum spinosum hypertrophy, and focal inflammatory cell infiltration [Figure 9].

## DISCUSSION

The promotion of bone regeneration, angiogenesis, and inflammation modulation through exosome therapy has been demonstrated in *in vivo* and *in vitro* models^[23]^. However, the effect of ASC-derived exosomes on lymphocyte phenotype in transplantation immunization has rarely been reported. The arena of organ transplantation continually seeks innovative strategies to regulate and optimize the post-transplant immune response. Within this framework, ASC exosomes have emerged as intriguing agents, offering the potential to modulate this response^[24,25]^. Our study delves into this potential, exploring the immunomodulatory effects of ASC-derived exosomes in a mouse skin graft model. The insights garnered have implications that may inform the broader landscape of organ transplantation.

Our research adds to the increasing body of evidence suggesting that ASC-derived exosomes can promote lymphocyte proliferation and modulate their phenotypes, potentially leading to an enhanced immune response following transplantation. These findings harmonize with prior studies examining diverse conditions like hepatocellular carcinoma and myocardial infarction^[26,27]^. This consistency across varied contexts underscores the broader potential of ASC-derived exosomes in organ transplantation scenarios. CD8+ T cells, recognized as cytotoxic T cells, are key mediators of transplant rejection^[28]^, whereas Treg cells function as immunomodulators, fostering immune homeostasis and tolerance following transplantation. In the mouse skin graft model, both ASCs and exosomes mitigated graft rejection by reducing the proportion of CD3+CD8+ T cells while enhancing the proportions of CD3+CD4+ T cells and Tregs.

The nuanced immunomodulatory roles of these exosomes, as evidenced by the promotion of Treg cells and simultaneous inhibition of CD8+ T cells and Th17 cells, illuminate a multifaceted tool for transplantation medicine. This hints at potential applications not only in skin grafting but possibly in other transplant scenarios, from kidney to liver transplants.

However, our observation of focal inflammatory cell infiltration after exosome injection serves as a word of caution. This underscores the importance of rigorous therapeutic optimization to harness the benefits while minimizing unintended immune activation. Striking the right balance will be imperative for the clinical application of exosome-based therapies.

Despite our promising insights, our study has its bounds. While this study explored the effects of mouse Adipose-derived stem cells (mASCs) and their exosomes on the immune phenotype of lymphocytes after skin grafting in mice, demonstrating that the exosome-treated group exhibited superior outcomes compared to the ASC-treated group, the precise mechanisms underlying these effects remain unclear. Based on our findings, both the exosome-treated and ASC-treated groups reduced the proportion of PD-1+ Treg cells, which could partly explain their role in prolonging graft survival. However, further studies are needed to elucidate the underlying mechanisms in greater detail. The exclusive use of a mouse skin graft model mandates additional research across other organ transplant models and, eventually, in human clinical settings. Moreover, unraveling the mechanisms driving the observed immunomodulatory effects will be crucial to refining and applying this therapeutic approach.

In conclusion, our study provides a promising window into the potential of ASC-derived exosomes as powerful agents in organ transplantation. As more insights emerge, the promise of exosome-based interventions in transplantation medicine could potentially reshape post-transplant care, offering new hope to transplant recipients worldwide.

## DECLARATIONS

### Availability of data and materials

All data generated or analyzed during this study are available in this article.

### Copyright

© The Author(s) 2025.
